# Pleiotropic Effects of Sodium-Glucose Cotransporter-2 Inhibitors: Renoprotective Mechanisms beyond Glycemic Control

**DOI:** 10.3390/ijms22094374

**Published:** 2021-04-22

**Authors:** Tomoaki Takata, Hajime Isomoto

**Affiliations:** Division of Gastroenterology and Nephrology, Faculty of Medicine, Tottori University, Yonago, Tottori 683-8504, Japan; isomoto@tottori-u.ac.jp

**Keywords:** sodium-glucose cotransporter, diabetic kidney disease, steatonephropathy, diabetic nephropathy, blood pressure, tubuloglomerular feedback, lipotoxicity, endoplasmic reticulum stress, mitochondria, uric acid

## Abstract

Diabetes mellitus is a major cause of chronic kidney disease and end-stage renal disease. However, the management of chronic kidney disease, particularly diabetes, requires vast improvements. Recently, sodium-glucose cotransporter-2 (SGLT2) inhibitors, originally developed for the treatment of diabetes, have been shown to protect against kidney injury via glycemic control, as well as various other mechanisms, including blood pressure and hemodynamic regulation, protection from lipotoxicity, and uric acid control. As such, regulation of these mechanisms is recommended as an effective multidisciplinary approach for the treatment of diabetic patients with kidney disease. Thus, SGLT2 inhibitors are expected to become key drugs for treating diabetic kidney disease. This review summarizes the recent clinical evidence pertaining to SGLT2 inhibitors as well as the mechanisms underlying their renoprotective effects. Hence, the information contained herein will advance the current understanding regarding the pleiotropic effects of SGLT2 inhibitors, while promoting future research in the field.

## 1. Diabetic Kidney Disease and the Treatment Strategy

Diabetic kidney disease (DKD) is a progressive kidney disease caused by diabetes mellitus and is a major public health concern worldwide. Approximately 40% of patients with diabetes develop chronic kidney disease (CKD) or end-stage renal disease [1,2]. Additionally, diabetes mellitus leads to end-stage renal disease in 39–46% of patients [3,4]. Kidney disease associated with diabetes typically presents as persistent albuminuria, resulting in diminished renal function. However, impaired renal function without albuminuria has also been reported [5]. Hence, the concept of DKD has emerged owing to this recent shift in the clinical presentation of kidney disease caused by diabetes mellitus [6]. That is, DKD is defined as CKD with diabetes-associated pathogenesis [7]. Considering that kidney dysfunction may latently progress in patients with DKD, it is important to improve the management of diabetes mellitus to prevent end-stage renal disease.

Persistent hyperglycemia causes inflammation, endothelial dysfunction, and oxidative stress in the kidneys and, thus, is partly associated with DKD progression [8,9,10]. In addition to traditional hypoglycemic agents and insulin, several agents targeting peptidyl hormones, such as dipeptidyl peptide IV and glucagon-like peptide-1, have recently been introduced for the treatment of hyperglycemia [11]. These therapeutic options have improved the management of hyperglycemia in patients with diabetes mellitus. However, as the pathogenesis of DKD is complex and multifactorial, a multidisciplinary approach is necessary to manage patients with CKD and diabetes. Accordingly, the Kidney Disease: Improving Global Outcomes (KDIGO) has recently proposed a clinical practice guideline for diabetes management in CKD patients [12], in which the fundamental approaches for all patients include glycemic and blood pressure control, as well as lipid management, whereas sodium-glucose cotransporter-2 (SGLT2) inhibitors and renin-angiotensin system (RAS) inhibitors are recommended for most patients.

RAS inhibitors have been used for decades and have demonstrated favorable effects in preventing CKD progression [13,14,15]. In contrast, SGLT2 inhibitors have only recently been developed for the treatment of diabetes to reduce glucose reabsorption in the renal proximal tubule. Clinical trials have shown that SGLT2 inhibitors prevent cardiovascular events in diabetic patients with or without CKD. Interestingly, SGLT2 inhibitors can also reduce cardiovascular events and prevent the progression to end-stage kidney disease in patients without diabetes [16]. In addition, in vitro and in vivo experiments have demonstrated that SGLT2 inhibitors exert renoprotective effects via various mechanisms independent of glycemic control. Thus, SGLT2 inhibitors are expected to play a central role in managing diabetes to overcome DKD. In this review, we summarize the clinical evidence related to SGLT2 inhibitors. We then focus on examining the potential mechanisms underlying their renoprotective effects in the context of glycemic control, blood pressure control, and lipid management; representing the fundamental approaches recommended in the KDIGO guideline. Of note, to achieve successful management of DKD, it is essential to compile the current relevant evidence, particularly those associated with the mechanisms of SGLT2 inhibitors.

## 2. Clinical Evidence on SGLT2 Inhibitors

To date, seven clinical trials have been conducted to investigate the effect of SGLT2 inhibitors on cardiovascular outcomes or CKD progression (Table 1 and Table 2) [16,17,18,19,20,21,22]. In the Empagliflozin Cardiovascular Outcome Event Trial in Type 2 Diabetes Mellitus Patients (EMPA-REG OUTCOME), empagliflozin significantly reduced cardiovascular events [17]. Empagliflozin also significantly reduced progression to macroalbuminuria, doubling of the serum creatinine level, initiation of renal replacement therapy, or death from renal disease in a post hoc analysis [23]. According to the Canagliflozin Cardiovascular Assessment Study (CANVAS) and the Multicenter Trial to evaluate the effects of dapagliflozin on the incidence of cardiovascular events (DECLARE-TIMI58), canagliflozin and dapagliflozin showed renoprotective effects [18,19]. These favorable effects of SGLT2 inhibitors on renal outcome were also demonstrated by a meta-analysis of these clinical trials [24]; however, patients included in these three studies were at low risk for renal failure, and the renal outcome was not validated as the primary outcome.

Meanwhile, the Canagliflozin and Renal Events in Diabetes with Established Nephropathy Clinical Evaluation (CREDENCE) trial recruited patients with overt albuminuria and an estimated glomerular filtration rate (eGFR) of 30–90 mL/min/1.73 m^2^ and included the following primary outcomes: composite outcome of end-stage renal disease, doubling of serum creatinine, and renal or cardiovascular death [20]. This trial confirmed that canagliflozin prevents renal outcomes in patients with advanced DKD and demonstrated that combination therapy with SGLT2 and RAS inhibitors is beneficial for preventing kidney disease progression. Interestingly, empagliflozin in the EMPA-REG OUTCOME and canagliflozin in the CREDENCE trial prevented cardiovascular or renal outcomes despite the small reduction in glycated hemoglobin level in many participants, suggesting that the pleiotropic effects of SGLT2 inhibitors reach beyond glycemic control [17,20].

Remarkably, the efficacy of SGLT2 inhibitors has been reported in patients without diabetes. For instance, the Dapagliflozin and Prevention of Adverse Outcomes in Heart Failure (DAPA-HF) and the Empagliflozin Outcome Trial in patients with chronic heart failure with reduced ejection fraction (EMPEROR-Reduced) trial targeted patients with heart failure regardless of diabetes status [16,21]. Although composite renal outcome, as the secondary endpoint, was not significantly reduced in DAPA-HF, cardiovascular events were significantly reduced by dapagliflozin regardless of concomitant diabetes [16]. In the EMPEROR-Reduced trial, empagliflozin prevented renal function decline in patients without diabetes. Taken together, these recent trials indicate that SGLT2 inhibitors exert protective effects on both cardiovascular and renal events, regardless of diabetes status.

## 3. Mechanisms Underlying the Renoprotective Effect of SGLT2 Inhibitors

**Figure 1:**
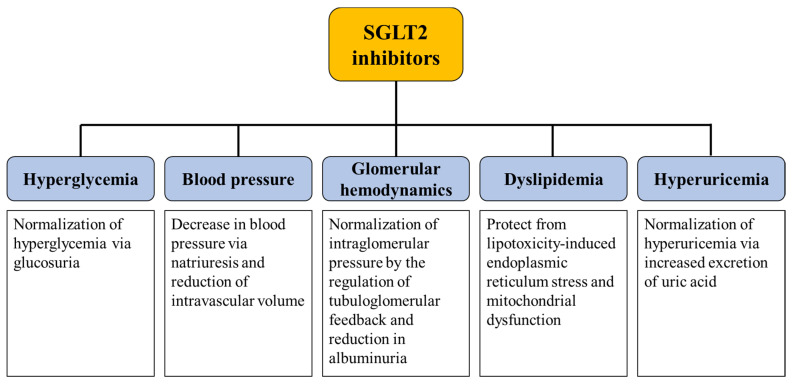
Summary of the potential mechanisms underlying the renoprotective effect of SGLT2 inhibitors. SGLT2 inhibitors potentially protect the progression of diabetic kidney disease through pleiotropic effects. SGLT2; sodium-glucose cotransporter-2.

Clinical evidence has indicated that the renoprotective effect of SGLT2 inhibitors depends not only on glycemic control but also on other unknown effects, the detailed mechanisms of which are not fully understood. We propose that SGLT2 inhibitors protect against the progression of kidney disease through pleiotropic effects (Figure 1). In this section, we summarize the mechanisms associated with the renoprotective effect of SGLT2 inhibitors, primarily focusing on the multidisciplinary approach recommended in the KDIGO guidelines.

### 3.1. Glycemic Control

Plasma glucose is freely filtered at the glomerulus and then reabsorbed by the proximal tubule. Under physiological conditions, urinary glucose excretion occurs when the filtered glucose level exceeds the maximum reabsorptive capacity of the proximal tubule. Luminal glucose filtered through the glomerulus is reabsorbed into the proximal tubular epithelial cells via the carrier-mediated transporters, SGLTs, and subsequently transported to the basolateral aspect of the epithelial cells through glucose transporters (GLUTs) 1 and 2. SGLTs are a family of glucose transporters encoded by *SLC5*. To date, seven SGLTs have been identified [25,26], among which SGLT1 and SGLT2 are the major isoforms investigated. SGLT1 is abundant in the small intestine and expressed in the kidney, whereas SGLT2 is exclusively expressed in the kidneys [25]. Both isoforms are localized in the apical membrane of the proximal tubule. Particularly, SGLT1 is expressed in the S3 segment of the proximal tubule, whereas SGLT2 is localized to the S1 segment. The maximum glucose reabsorption capacity of the S1 segment is higher than that of the S2 and S3 segments [27]. Therefore, the selective inhibition of SGLT2 causes glucose overload that exceeds the reabsorptive capacity of downstream segments, leading to glucosuria. Although compensatory upregulation of SGLT1 exists [28], SGLT2 inhibition increases net urinary glucose excretion to approximately 200–300 kcal of daily energy loss [29]. Thus, dual inhibitors of SGLT1 and 2 may be more effective in terms of glycemic control. However, their efficacy and safety must be confirmed in clinical trials. In addition, because SGLT1 is distributed in the intestine, gastrointestinal symptoms, such as diarrhea and dehydration, may occur [30].

### 3.2. Glomerular Hemodynamics, Natriuresis, and Tubuloglomerular Feedback

The current paradigm for the renoprotective mechanism of SGLT2 inhibitors is considered to have an effect on renal hemodynamics, including natriuresis and tubuloglomerular feedback [31,32]. Diabetes causes glomerular hypertension by impairing the renal hemodynamic autoregulation system. This involves the dilatation of the arteriole connecting to the glomeruli, with a more pronounced effect on the afferent arteriole than the efferent arteriole [33]. The dissociation of this vasodilatory change in afferent and efferent arterioles causes a proportionally greater increase in efferent arteriolar resistance and a decrease in afferent arteriolar resistance, resulting in increased intraglomerular pressure. Furthermore, nephron loss, that accompanies the progression of kidney disease, causes compensatory hyperfiltration in the remaining nephrons. This compensatory increase in single nephron GFR observed in diabetic patients subsequently leads to podocyte loss and albuminuria, thereby accelerating kidney disease progression. Therefore, a reduction in intraglomerular pressure is beneficial for renoprotection. RAS inhibitors induce vasodilation of the efferent arteriole, which attenuates kidney disease progression [34]. Although the reduction in intraglomerular pressure by RAS inhibitors was reflected in the initial decrease in GFR, long-term GFR levels were stable in diabetic patients treated with RAS inhibitors [35].

Glomerular arteriolar resistance is finely adjusted via tubuloglomerular feedback [36]. Urinary flow and the NaCl concentration in the tubular lumen at the end of the loop of Henle are sensed by the macula densa through the Na^+^, K^+^, Cl^-^ cotransporter isoform 2 (NKCC2) and the renal outer medullary potassium (ROMK) type K+ channels. The transition segment of the loop of Henle to the distal convoluted tubule lies adjacent to the afferent and efferent arterioles of the same nephron, and the increased delivery of NaCl to this segment triggers the vasoconstriction of afferent arterioles with relative relaxation of the efferent arteriole, leading to reduced single nephron GFR [31]. Under diabetic conditions, augmented Na reabsorption, coordinated with increased glucose reabsorption via SGLT1 and 2, reduces NaCl delivery to the macula densa and increases GFR via tubuloglomerular feedback [37]. Considering that SGLT2 blockade not only leads to glucosuria, but also natriuresis, SGLT2 inhibitors can achieve NaCl delivery to the distal portion of the nephron. This natriuretic effect of SGLT2 inhibitors regulates tubuloglomerular feedback, resulting in decreased intraglomerular pressure.

**Figure 2:**
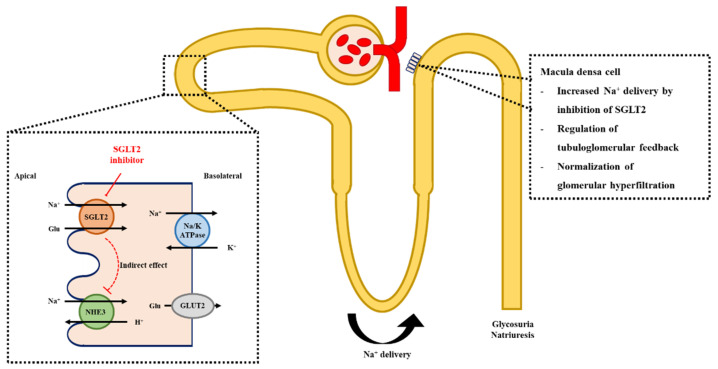
Mechanism of SGLT2 inhibition and the regulation of tubuloglomerular feedback. Inhibition of SGLT2 indirectly suppresses the activity of NHE3, leading to increased Na^+^ delivery to the distal part of the nephron. The elevation of Na^+^ concentration and increased luminal flow activate the tubuloglomerular feedback and decrease intraglomerular pressure. SGLT2; sodium-glucose cotransporter-2, NHE3; Na^+^/H^+^ exchanger 3.

Indeed, SGLT2 inhibition causes the compensatory upregulation of SGLT1 [28,38]. The reabsorptive capability of sodium differs between SGLT1 and 2; that is, SGLT2 has a coupling stoichiometry of 1 Na:1 glucose, whereas SGLT1 has 2 Na:1 glucose [25]. Therefore, the enhanced sodium reabsorption coordinated with glucose reabsorption through SGLT1 may theoretically diminish, or even invert, the natriuretic effect of SGLT2 inhibition. A recent investigation suggested the involvement of Na^+^/H^+^ exchanger 3 (NHE3), which colocalizes with SGLT2, in SGLT2 inhibitor-mediated natriuresis. In fact, within animal studies, SGLT2 inhibitors were reported to alter NHE3 to a natriuretic profile via an indirect effect through intracellular glucose metabolism and changes in osmolarity, resulting in reduced sodium reabsorption at the proximal tubule [38,39]. This resulted in decreased net reabsorption of sodium in the proximal tubular segment via SGLT2 inhibition. In patients with diabetes, the administration of SGLT2 inhibitors increased urinary sodium excretion [40,41]. These natriuretic effects, together with the modulation of tubuloglomerular feedback after SGLT2 inhibition, may depend on the regulation of NHE3 activity (Figure 2).

Changes in single nephron GFR have been investigated in a micropuncture study in diabetic rats. Results for which indicate that SGLT2 inhibition increases the chloride concentration in the early distal tubule, while reducing single nephron GFR [42]. A similar phenomenon has been observed in diabetic mice, i.e., reduction in single nephron GFR and contraction of the afferent arteriole after empagliflozin injection [43]. In contrast to the reduction in single nephron GFR observed in the type 1 diabetes model, administration of dapagliflozin did not impact glomerular size in BSA-injected type 2 diabetes mouse model [44]. Meanwhile, in db/db mice treated with luseogliflozin, the glomerular volume increased [45]. Therefore, the effect of SGLT2 inhibition on glomerular or kidney size depends on the type of diabetes and stage of kidney disease. Kidney length increased after 6 months of dapagliflozin treatment in patients with type 2 diabetes [46]. Because a single nephron GFR is defined by the relative flux between the afferent and efferent arterioles, single nephron GFR may not directly correlate with glomerular size. Kidney volume, particularly cortical volume where glomeruli are distributed, is positively correlated with GFR and predicts kidney disease progression [47,48]. Further investigation is required to determine the association between glomerular hemodynamic changes and glomerular or kidney size after SGLT2 inhibition.

Clinical trials have shown that SGLT2 inhibitors affect GFR in patients with diabetes. Similar to RAS inhibitors, SGLT2 inhibitors cause an initial decrease in the acute phase, followed by sustained renal function during the chronic phase [20,22]. In addition to GFR changes, according to the CREDENCE trial, canagliflozin can also reduce urinary albuminuria and prevent the progression of micro/macroalbuminuria [20].

### 3.3. Protection from Lipotoxicity

**Figure 3:**
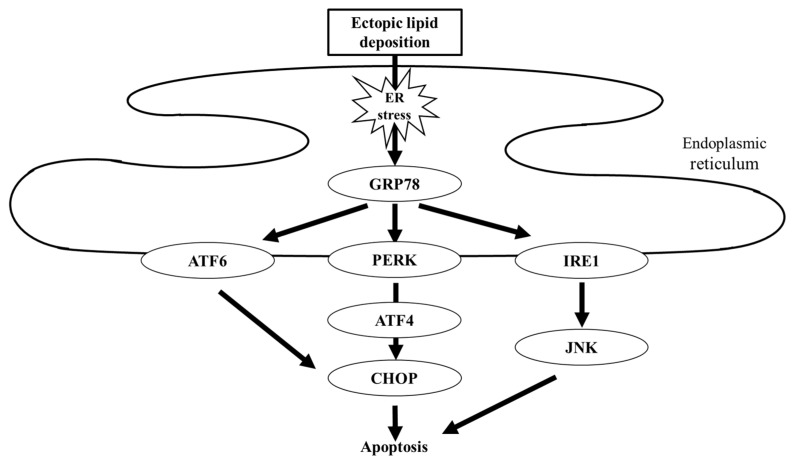
Schematic of lipotoxicity-related ER stress. Ectopic lipid deposition induces ER stress. Excessive ER stress is sensed by three different mediators, namely ATF6, PERK, and IRE1, resulting in cellular apoptosis. GRP78, glucose-regulated protein-78; ATF, activating transcription factor; PERK, PKR-like endoplasmic reticulum kinase; IRE1, inositol requiring 1; and CHOP, C/EBP homologous protein.

Dyslipidemia, characterized by hyperlipidemia, hypercholesterolemia, and hypertriglyceridemia, is an important therapeutic target for patients with DKD [12]. Abnormal lipid metabolism, as evidenced by quantitative and qualitative changes in lipoprotein composition, promotes the progression of kidney disease in DKD [49]. Similar to the liver, the major organ affected in steatosis, dyslipidemia accelerates ectopic lipid deposition in the kidney [50]. Ectopic lipid droplets in the glomeruli and tubular cells induce inflammation, ROS production, and endoplasmic reticulum (ER) stress [51,52,53], which plays a central role in the progression of kidney disease in steatonephropathy. ER stress-mediated cellular apoptosis occurs via induction of glucose-regulated protein-78 (GRP78), a master regulator of ER stress. Three different pathways have been identified as downstream of GRP78-mediated apoptosis, in which the activating transcription factor 4 (ATF4) and C/EBP homologous protein (CHOP) act as mainstream intermediates (Figure 3) [54,55]. Recent investigations have suggested that SGLT2 inhibitors can attenuate renal tubular injury caused by ER stress. In a mouse model of induced renal steatosis, ipragliflozin reduced lipid deposition in renal tubules, which was associated with decreased expression of GRP78 and CHOP and cellular apoptosis [56]. Similarly, dapagliflozin ameliorated ER stress-mediated cell death in diabetic mice and in proximal tubular cells through the GRP78-ATF4-CHOP pathway [57,58].

Mitochondrial dysfunction is another pathophysiology that is closely associated with lipotoxicity. Mitochondria are intracellular organelles that play a pivotal role in maintaining the energy supply via fatty acid β-oxidation. Fatty acids are the most powerful substrates for adenosine triphosphate production, while the kidney is one of the major organs with a high demand for energy to maintain its metabolic activities. In particular, the renal proximal tubule, which contains abundant mitochondria, primarily relies on fatty acids as an energy source [59]. Therefore, proximal tubular cells are vulnerable to dysregulated mitochondrial fatty acid oxidation. Excess fatty acids induce intracellular accumulation of free fatty acids and triglycerides that induce ROS production in the mitochondria [60]. Disturbed mitochondrial fatty acid oxidation causes energetic failure of proximal tubules [61]. Furthermore, mitochondrial dysfunction results in decreased lipid oxidation followed by lipid accumulation, which leads to a vicious cycle of mitochondrial dysfunction and lipotoxicity [62].

SGLT2 inhibitors are expected to improve mitochondrial energetics in diabetic kidneys. In a mouse model of DKD, canagliflozin was shown to ameliorate mitochondrial fatty acid oxidation and improve mitochondrial biogenesis and function [63]. Another SGLT2 inhibitor, ipragliflozin, restores mitochondrial morphology by maintaining the expression of *Opa1* and *Mfn2*, the key molecules for mitochondrial fusion, in mice fed a high-fat diet [64]. Although the full mechanism is under debate, SGLT2 inhibitors indisputably exert renoprotective effects by ameliorating cellular stress or improving metabolism in intracellular organelles, including the ER and mitochondria.

### 3.4. Uric Acid Control

Hyperuricemia is associated with an increased risk of DKD progression in patients with type 1 or type 2 diabetes [65]. Uric acid induces inflammation, ROS production, and endothelial dysfunction [66], which accelerate kidney disease and increase the risk of cardiovascular disease in patients with CKD. Therefore, uric acid is a target for preventing the progression of kidney disease [67,68]. Xanthine oxidase inhibitors or urate transporter 1 inhibitors have been used as standard therapies for hyperuricemia. SGLT2 inhibitors are expected to be an alternative option for the treatment of hyperuricemia by increasing the urinary excretion of uric acid. A meta-analysis of randomized controlled trials in patients with type 2 diabetes showed that SGLT2 inhibitors lowered serum uric acid levels from baseline [69]. SGLT2 inhibitors have also been reported to reduce the risk of gout in patients with type 2 diabetes [70]. The effect of SGLT2 on the downregulation of serum uric acid levels is associated with reduced reabsorption of uric acid in the kidneys. Uric acid filtered through the glomerulus is primarily reabsorbed in the proximal tubule. The glucose transporter 9 (GLUT9), encoded by *SLC2A9*, which causes renal hypouricemia, is reportedly involved in SGLT2 inhibitor-mediated uric acid excretion. GLUT9 has two subtypes: GLUT9b localizes in the apical membrane and acts as a transporter for glucose and uric acid, whereas GLUT9a is distributed in the basolateral membrane of the S1 segment of the proximal tubule [71,72]. Therefore, an increase in intraluminal glucose after SGLT2 inhibition leads to increased reabsorption of glucose via GLUT9b, thus disturbing the reabsorption of uric acid [73].

## 4. Conclusions

SGLT2 inhibitors were originally developed for glycemic control in patients with diabetes mellitus. As they have shown promising benefits in nearly all clinical trials, SGLT2 inhibitors are expected to serve as the “magic bullet” for patients with or without diabetes. Increasing clinical evidence has revealed the renoprotective effects of SGLT2 inhibitors primarily via glycemic control and hemodynamic regulation. Here, we propose that SGLT2 inhibitors also exert pleiotropic effects related to the fundamental approach for treatment of patients with or without diabetes and CKD. Thus, SLGT2 inhibitors can be administered to a broad range of patients with a risk of CKD. Moreover, although the physiological and pharmacological mechanisms of SGLT2 inhibitors are not fully characterized, current progress identifying the roles played by SGLT2 inhibitors also supports their use for treatment of DKD.

## Figures and Tables

**Table 1:** Characteristics of patients in SGLT2 inhibitor clinical trials.

Clinical Trial	Year	Drug (Dose)	*N*	Age (Years)	Median Follow-Up Period (Years)	CVD	DM	Mean eGFR (mL/min/1.73 m^2^)
EMPA-REG	2015	Empagliflozin (10 mg, 25 mg)	7020	63.1	3.1	100%	100%	74.1
CANVAS	2017	Canagliflozin (100 mg, 300 mg)	10142	63.3	2.4	65.6%	100%	76.5
DECLARE-TIMI 58	2019	Dapagliflozin (10 mg)	17160	63.9	4.2	40.6%	100%	85.2
CREDENCE	2019	Canagliflozin (100 mg)	4401	63.0	2.6	50.40%	100%	56.2
DAPA-HF	2019	Dapagliflozin (10 mg)	4401	66.3	1.5	100%	41.8%	65.8
EMPEROR-Reduced	2020	Empagliflozin (10 mg)	4401	66.8	1.3	100%	49.8%	62.0
DAPA-CKD	2020	Dapagliflozin (10 mg)	4401	61.9	2.4	37.4%	67.5%	43.1

N, number; CVD, cardiovascular disease; DM, diabetes mellitus; eGFR, estimated glomerular filtration rate.

**Table 2:** Renal outcome in clinical trials.

Clinical Trial	Definition of Renal Outcome	HR (95% CI)
EMPA-REG	Progression to macroalbuminuria, doubling of sCr, initiation of RRT, or death from renal disease	0.61 (0.53–0.70)
CANVAS	40% reduction in eGFR, requirement for RRT, or death from renal causes	0.60 (0.47–0.77)
DECLARE-TIMI 58	40% decrease in eGFR, ESRD, death from renal or cardiovascular causes	0.76 (0.67–0.87)
CREDENCE	ESRD, doubling of the sCr, or death from renal or cardiovascular causes	0.70 (0.59–0.82)
DAPA-HF	50% decline in the eGFR, ESRD, or renal death	0.71 (0.44–1.16)
EMPEROR-Reduced	Hemodialysis, renal transplantation, or profound sustained reduction in eGFR	0.50 (0.32–0.77)
DAPA-CKD	Sustained decline in the eGFR, ESRD, or death from renal or cardiovascular causes	0.61 (0.51–0.72)

HR, hazard ratio; CI, confidence interval; sCr, serum creatinine; RRT, renal replacement therapy; ESRD, end-stage renal disease.

## Data Availability

Not applicable.
